# Prediction of Neurodevelopmental Outcomes in Very Preterm Infants: Comparing Machine Learning Methods to Logistic Regression

**DOI:** 10.3390/children11121512

**Published:** 2024-12-12

**Authors:** Jehier Afifi, Tahani Ahmad, Alessandro Guida, Michael John Vincer, Samuel Alan Stewart

**Affiliations:** 1 Division of Neonatal Perinatal Medicine, Department of Pediatrics, Dalhousie University, Halifax, NS B3K 6R8, Canada; michael.vincer@iwk.nshealth.ca; 2 Department of Diagnostic Imaging, Dalhousie University, Halifax, NS B3K 6R8, Canada; tahani.ahmad@iwk.nshealth.ca (T.A.); alessandro.guida@dal.ca (A.G.); 3 Department of Community Health & Epidemiology, Dalhousie University, Halifax, NS B3H 1V7, Canada; sam.stewart@dal.ca

**Keywords:** machine learning, predictive modeling, preterm infants, neurodevelopment

## Abstract

Purpose: Is machine learning (ML) superior to the traditionally used logistic regression (LR) in prediction of neurodevelopmental outcomes in preterm infants? Objectives: To develop and internally validate a ML model to predict neurodevelopmental impairment (NDI) in very preterm infants (<31 weeks) at 36 months corrected age, using clinical predictors. Methods: A retrospective cohort of very preterm infants (23^0–^30^6^ weeks) born between January 2004 and December 2016 in Nova Scotia, Canada. Survivors with neurodevelopmental assessment at 36 months corrected age were included. The study sample was randomly split (80:20) into a development and testing datasets. We compared four methods: LR, elastic net (EN), random forest ensemble (RF) and gradient boosting (XGB), in relation to discrimination (AUC), calibration, and diagnostic properties. Results: Of 811 eligible infants, 663 were included (mean gestational age 28 weeks, mean birth weight 1137 g and 52% male). Of those, 195 (29%) developed NDI and 468 (71%) did not. On internal validation using the testing dataset, all four models provided good discrimination of NDI with comparable AUC. RF was superior to the other three methods with a higher AUC (0.79 vs. 0.74, 0.74, and 0.73 for XGB, EN and LR, respectively), but all models have overlapped CIs. Conclusions: In this population-based cohort of very preterm infants, RF was superior to conventional LR in prediction of NDI at 3 years corrected age. Accurate prediction of preterm infants at risk of NDI enables early referrals for intervention programs and resources allocation toward those who are most likely to benefit.

## 1. Introduction

Despite improved survival of preterm infants, neurodevelopmental impairment (NDI) remains a considerable long-term morbidity [1,2,3]. The rates of NDI range from 15–40% in high-resource settings and even higher in developing countries [2]. Definition of NDI encompasses motor, language, and cognitive deficits as well as learning and behavioral problems [1,2,3]. Accurate identification of preterm infants at risk of NDI enables referral to targeted early interventions, with the potential to improve functional outcomes and quality of life [4]. Accurate and early prediction of NDI is also extremely valuable for counselling parents at risk of preterm birth, specifically when deciding about continuing intensive care or performing emergent interventions [5]. For health care providers, prediction models will assist in providing individualized targeted care and in streamlining the healthcare resources allocation (rehabilitation, family resources, and social support) [6].

Conventional logistic regression (LR) had been traditionally used for prediction of binary outcomes in neonatal research, being simple and explanatory(interpretable). However it has many drawbacks; it assumes a linear relationship between the predictors and the outcome and performs poorly if complex or non-linear dataset, with high-order interactions between predictors or when large number of predictors relative to the sample size [7]. There is a multitude of methods in the statistical and machine-learning (ML) world that can be used for this type of prediction problem [6].

The role of regularization or penalized regression is increasingly emphasized in risk prediction. It imposes a penalty to LR for having too many predictors and automatically performs feature selection, by shrinking coefficients of less important variables to zero, thus keeping only the most contributive predictors in the model. Elastic Net regression (EN) is the current state-of-the-art penalized regression, particularly for large datasets data with many correlated predictors, where feature selection and regularization are needed to prevent overfitting [8]. Elastic Net provides a balance between ridge and LASSO regression methods, thus helping with issues related to multicollinearity and sparcity [8]. However, like LR it assumes linear relation between predictors and outcome.

Recently, ML may perform better than conventional statistical methods due to their adaptability to high-dimension data with multiple variables and ability to handle complex and non-linear relation between predictors and outcome [9]. Although considered as “black-box”, being non-explanatory, and requires complex computation, Random Forests (RF) and Gradient Boosted Trees (XGB) are two popular ML methods that could improve prediction over LR [10,11]. They also provide feature importance plots that give insights into the relative importance of different variables in the prediction model. Supplementary Table S1 compares and contrasts these four prediction methods.

The objective of this study was to develop and conduct internal validation of a model to predict NDI in very preterm infants, using clinical predictors readily available in medical records. We examined a combination of statistical (LR, EN) and ML methods (RF, XGB), and compared their predictive accuracy to determine the optimal model of NDI in these infants.

**Hypothesis** **H1.** *In very preterm infants (<31 weeks’ gestation), EN and ML methods (RF and XBG) will outperform the traditionally used LR in predicting NDI at 36 months corrected age (CA).*

## 2. Methods

### 2.1. Study Design

This is a population-based study of a large cohort of very preterm infants in Nova Scotia, Canada. We compared four methods for prediction of NDI: (conventional LR, EN, RF and XGB) and reported their predictive performance in terms of discrimination, calibration, and diagnostic properties. The population sample was split into an 80% training dataset to develop the models and a 20% testing dataset to validate them, stratified on the outcome variable to ensure similar event rates in both datasets. The study was approved by the IWK Research Ethics Board (Project #:1025476).

### 2.2. Population

All very preterm infants (23^0^–30^6^ weeks’ gestation) born between January 2004 and December 2016 were eligible. We excluded those with congenital anomalies, those who received palliation at birth, those who died before 36 months CA and those with missing outcome data. Survivors with complete neurodevelopmental assessment at 36 months CA were included in the analysis.

### 2.3. Data Source and Provincial Perinatal Follow-Up Program

All surviving very preterm infants whose mothers reside in Nova Scotia are routinely followed at the Provincial Perinatal Follow-Up Program (PFUP) for up to 3 years of age. Neurodevelopment is determined based on standardized validated tests conducted by an experienced multidisciplinary team at 4-, 8-, 18- and 36-months CA, with additional follow-up if abnormalities are detected at any visit. Assessment included neuromotor examination for diagnosis of cerebral palsy (CP), Bayley Scales of Infant and Toddler Development (BSITD-III) [12], ophthalmologic and auditory assessment. Based on this detailed assessment, very preterm children were divided into two groups: those with NDI and those without NDI.

The AC Allen Perinatal Research Database collects clinical data on all very preterm infants followed in the PFUP, including maternal sociodemographic, prenatal, perinatal, and postnatal data (predictors), and neurodevelopmental assessment (outcome). The database also collects radiologic reports of routine sequential cranial ultrasounds (CUS) performed on all very preterm infants on days of life 4, 14, 42 and at term-equivalent age. Severe neurologic injury on CUS was defined as any of the following: severe intraventricular hemorrhage (grade 3 or 4) [13], cystic periventricular leukomalacia, significant ventricular dilatation or non-punctate cerebellar hemorrhage [14,15,16]. Unremarkable CUS indicates the absence of all the above-mentioned abnormalities on routine CUS screening.

### 2.4. Outcome

The primary outcome is to develop a prediction model of NDI at 36 months CA, defined as any of the following: CP, BSITD-III scores < 1 SD below the mean in any domain (cognitive, motor, language), hearing or visual impairment.

### 2.5. Candidate Predictors

All candidate predictors were abstracted from medical records at hospital discharge, including clinical and radiologic variables as described. We selected 73 candidate predictors from the database based on content expertise and literature. Of these variables, 11 were removed because of singular data patterns (>95% of infants with a single value) and 1 variable was dropped for significant missingness (>15%). There were 17 variables that had some additional missing values; to avoid losing a significant number of predictors we imputed the missing values using chained equations. A complete list of all 73 candidate predictors is provided in Supplementary Table S2.

### 2.6. Statistical Analysis

Descriptive characteristics of children with and without NDI were summarized as proportions, mean (standard deviation) or median (interquartile range), as appropriate. The population characteristics were compared between the study groups using fisher exact test for categorical variables and Mann Witney test for continuous variables.

Four analytic models were developed, and all models were built on the same set of prediction variables listed in Supplementary Table S2. For LR, there were no hyperpa-rameters, but the number of predictors relative to the sample size presented a problem, so univariate pre-screening was performed. All candidate variables were fit individually with the outcome of NDI, and predictors with a *p*-value <0.05 were included in the final model resulting in 37 candidate features included in the model. For EN, two hyperparameters (alpha and lambda) were estimated using 5-fold cross-validation with a 10 × 10 grid space search on each [8]. The EN model was built using an alpha value of 1 and a lambda value of 0.023. Random Forest had minor hyperparameters (1000 trees, 25 candidate variables per split and 55% (25/45) feature sampling rate) that were set based on pre-liminary exploration of data [10]. For XGB, we used the xgbTree library in R which has several hyperparameters that were estimated using the same grid-based strategy and 5-fold cross-validation [11]. The XGB model had the following hyperparameters: 600 rounds of sampling, forced tree depth in [3, 7], eta = 0.3, gamma = 0.7, and 80% sampling rate.

The presence of class imbalance (30% NDI vs. 70% no NDI) poses a problem, particularly with classification performance favoring the majority class (no NDI). This may result in poor predictive performance of the minority class. To avoid this problem, class weights were set according to the baseline distribution of NDI in the population sample.

Receiver Operator Characteristic (ROC) curves were calculated for all four models to present graphically and for the calculation of the area under the curve (AUROC) for each model with their 95% confidence intervals (CIs). Using the ROC, the optimal cut point for each model was calculated using the Youden’s Distance [17]. The performance of these prediction models was compared in relation to discrimination (AUROC), calibration and diagnostic properties (sensitivity, specificity, positive and negative predicted values (PPV, NPV) and accuracy). To investigate the agreement/disagreement between the models an ensemble predictor was constructed, where each model contributes a single vote to a combined predictor.

For all 4 models, we evaluated the variable importance to identify the most influential features. For LR and EN, variable importance was calculated as the absolute value of the test-statistic for the coefficients. For RF and XGB variable importance was calculated as the total gain in GINI coefficients across all trees. Of note, RF calculates variable importance at a variable level, while the other three methods report it at a coefficient level. All importance metrics were scaled such that the highest importance is 100, per model [18]. The statistical analysis was performed using R version 4.2.1 with the packages caret [18], randomForest [10], xgboost [11], and glmnet [8].

## 3. Results

**Figure 1:**
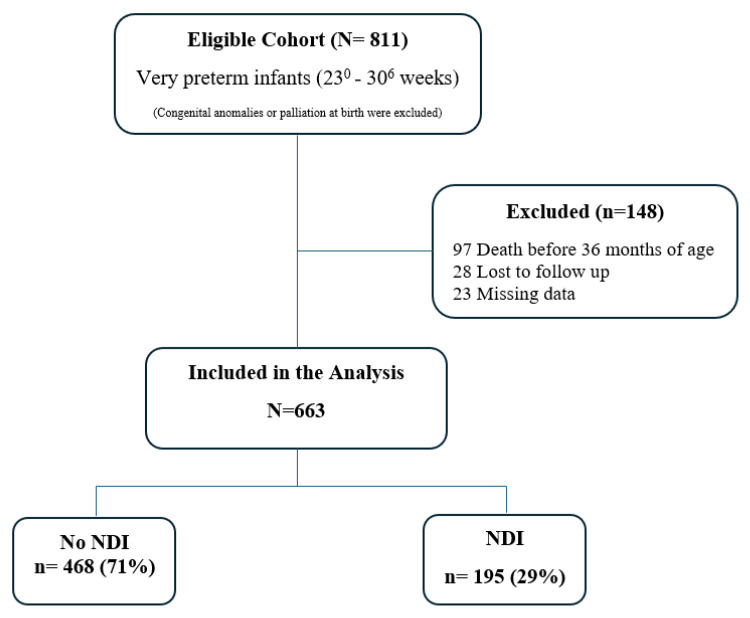
Population Flow Chart.

Over the study period, 811 eligible very preterm infants were born. Of those, 148 infants were excluded (23 with missing outcome data, 97 who died before 36 months CA, and 28 were lost to follow up) resulting in 663 (82% of the eligible cohort) being included in the analysis (Figure 1). Their mean gestational age was 28.1 (1.7 SD) weeks, mean birth weight was 1137 g (316 SD) and 52% were male. There were 195 infants (29%) that developed NDI and 468 who had no NDI (71%) in this cohort (Figure 1).

Table 1 describes the population characteristics and compares the candidate predictors between the study groups based on NDI status and summarizes the variables that were eventually removed from the model.

**Figure 2:**
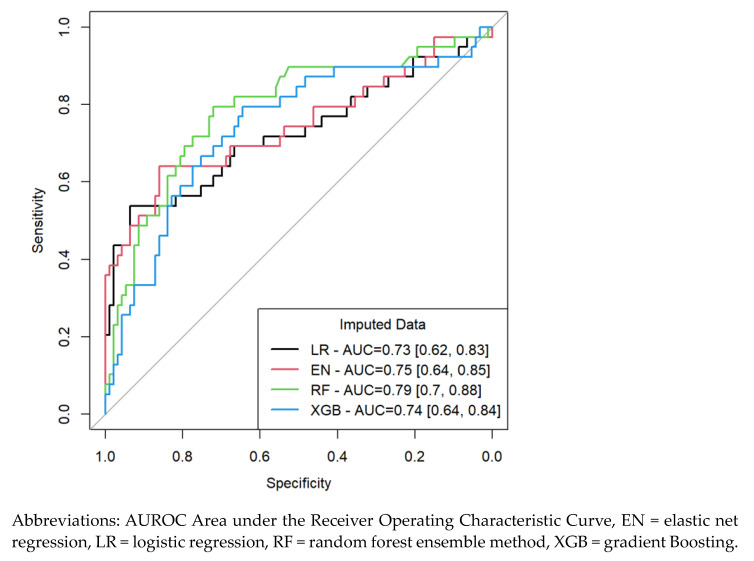
The Area Under the Receiver Operating Characteristic Curve (AUROC) of the Four Prediction Models of Neurodevelopmental Impairment in Very Preterm Infants.

On internal validation using the testing dataset, all four methods provided comparable discriminative ability, but machine learning methods (RF and XGB) and EN were superior to conventional LR in prediction of NDI in very preterm infants (Figure 2). RF was superior to the other three methods with a higher AUROC (0.79 vs. 0.74, 0.74, and 0.73 for XGB, EN and LR, respectively), but all models have overlapped CIs (Figure 2).

**Figure 3:**
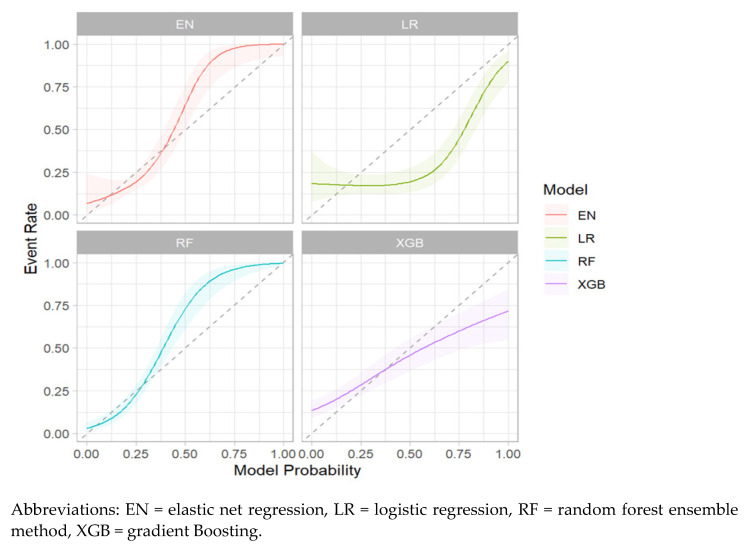
Calibration Plots of the Four Prediction Models of Neurodevelopmental Impairment in Very Preterm Infants.

Figure 3 describes the calibration plots for the four prediction models. As shown, neither plot revealed significant differentiation between the methods (Figure 3).

The discrimination, accuracy, and diagnostic properties of the four prediction models are shown in Table 2. RF and XGB had optimal cut points of ≥0.25 and ≥0.15 respectively, producing accuracies of 74% and 69%. Both models ended with good sensitivity of 79% and high NPV of 89%. On the other hand, LR and EN had optimal cut points of ≥0.72 and ≥0.31, respectively, and had accuracies of 82% and 79%. These two models provided high specificity (93% and 86%) and NPV (83% and 85%).

The ensemble predictor is shown in Table 3. When each model contributes a single vote to a combined predictor, the accuracy did not improve significantly; of the 58 test set subjects that received no votes from any of the models, 5 of them (9%) had NDI, and conversely of the 25 subjects that received a vote from all 4 models, only 20 of them (80%) had NDI. A complete agreement ensemble had an overall accuracy of 82%, with sensitivity of 0.51 and specificity of 0.95.

The Variable importance of all four models is shown in Table 4 describing the ten most important predictors for each model. Receipt of neonatal resuscitation (chest compression or epinephrine) and white matter disease (defined as periventricular leukomalacia or porencephaly) were the most important predictors of NDI using LR and EN, whereas maternal age and z-scores of birth weight were the most important predictors using ML (RF and XGB).

## 4. Discussion

In this study, we used a novel method to develop and internally validate prediction models of NDI in very preterm infants at 36 months CA. We compared penalized regression (EN) and ML (RF and XGB) to the conventional LR and showed superior performance of ML methods and EN over LR in prediction of NDI in this population. Of the four prediction methods, RF performed the best [AUC 0.79, 95% CI (0.70–0.88)] while LR performed the worst [AUC 0.73, 95% CI (0.62, 0.83)], but all four methods had significant overlap in their AUC and CIs. The study also identified clinical predictors of NDI and provided insight into ML variable importance ranking of these predictors. Except for AUC, one should caution the interpretation of the diagnostic properties of the developed models as they depend on the selected cut-off and would result in different values for the same sample, if a different cut-off was used.

In this cohort, 88% of very preterm infants survived, but almost one in three survivors developed NDI. With improved survival of preterm infants at highest risk for NDI, research that focuses on developing early and accurate prediction models of long-term outcomes in this population is very valuable for patients, their families and healthcare providers. To the best of our knowledge, this study is one of the first population-based studies to use ML for prediction of NDI in very preterm infants, using clinical predictors that can be readily abstracted from patient records.

Most longitudinal studies in preterm population reported measures of association and not prediction. Over the last two decades, ML has been increasingly used in health research for prediction of disease risk and prognosis [6,19,20,21,22,23]. Machine learning offers a theoretical advantage over LR as it can handle complex, non-linear relation between predictors and outcome, can identify higher-order interactions between predictors, and does not require explicit model specification. However, they are more difficult to interpret, as they are non-explanatory as opposed to conventional statistical methods. Some progress has been made in this area, with concepts like variable importance providing insight into the variables that the models are using in their decision-making process. In Table 4, we reported the ten most important predictors for all four models and majority of the included predictors have established physiologic plausibility to the outcome of NDI. Notably, there was similarity in predictors of the tree-based models (RF and XGB) sharing 7 out of the 10 predictors. Similarly, LR and EN shared 6 of the 10 predictors, and this is not surprising given the similarity between the two methods. Of interest, the focus in the tree-building models is on continuous predictors (4 out of 10) compared to LR and EN (1 out of 10) being continuous predictors. This probably reflects the ability of ML to detect non-linear trends, which is most prominent when investigating continuous predictors, and will investigate all possible splits, rather than assuming a linear relationship as LR and EN.

Use of ML for prediction of long-term outcomes in preterm infants is relatively a new field with lots of opportunities to be explored. Recently, two reviews investigated applying ML to structural or functional magnetic resonance imaging for prediction of NDI in preterm infants [20,21]. The included studies showed promising results, but identified opportunities for improvement in future models, both in methodology and reporting. However, these reviews were limited by small sample size (range 59–368 preterm infants) and lack of clinical predictors. To our knowledge, three recent studies (1 single center and 2 large cohorts) applied ML to clinical variables for prediction of NDI in extreme preterm infants at 2 years of CA (23–25). These studies reported AUC ranging from 0.67 to 0.92. However, this current study is not comparable to previous published ML research for the following reasons; First, two out of three studies examined the composite outcome of death or NDI as the primary outcome compared to NDI among survivors in our analysis [23,24]; Second, one study focused on the continuous definition of NDI based on BSITD scores [24], whereas we examined the binary classifier of NDI; Third, study groups are not comparable with some studies focusing on severe NDI, thus including those with no NDI as well as those with moderate NDI as the comparable group with favorable outcome [24]; Finally, two studies used early (first 2 postnatal weeks) and late (at 6 and 12 months CA) predictors [23,25] rather than targeting hospital discharge as the prediction point in our analysis. In our cohort, the prediction models achieved good but not excellent discrimination and classification. As the ensemble approach demonstrated, even at the aggregate level, the differentiation between children with and without NDI is not without limitations. This could be probably due to the nature of NDI as a complex outcome, so some predictors will perform better for certain components of this outcome (periventricular leukomalacia for CP). Future research is needed to investigate other potential features that could booster prediction of outcomes in preterm population.

### Strengths and Limitations

This study has several strengths. First, the low attrition bias with low loss to follow up rate (82% of the birth cohort and 93% of survivors were included). Second, being a population-based cohort, it is more generalizable than existing literature often derived from small cohorts or limited to only a subgroup of high-risk preterm infants (with abnormal neuroimaging antecedent to NDI). Third, the validity of the developed model as the PFUP database kept consistent definition of NDI over the study period and standardized assessment and timing of predictors and outcome. Finally, we followed the recommended standards for development and reporting of prediction models using AI methods [25].

This study is not without limitations. Being a retrospective study, we may have excluded potential predictors which would affect the selected predictors and the predictive performance. Some known predictors of NDI (such as race/ethnicity, illness severity scores) were not included in the analysis, because of inconsistency and missingness that would have impacted the internal validity of the study. The fact that most of the population in Nova Scotia are Caucasians would limit the generalizability of the findings to other populations with different ethnic groups or race-related determinants of health.

## 5. Conclusions

In this population-based cohort of very preterm infants, we developed and internally validated a prediction model of NDI using machine learning. We compared different prediction methods and showed superior performance of random forest ensemble over conventional logistic regression, which is traditionally used in neonatal research. Accurate risk prediction of preterm survivors would help resources allocation toward those who are most likely to benefit.

## 6. Implications for Clinical Practice and Future Research Directions

To our knowledge, this is one of few recent studies in literature that applied ML to clinical data to predict long-term outcomes in preterm population. Our study provides insight to fill the knowledge gap in this area. Prediction models of neurodevelopmental outcomes are important for healthcare providers for prognostication and to guide resource allocation towards those who are most likely to benefit. If external validation of the developed model to predict NDI in preterm population showed good predictive accuracy, the model can be used as an adjunct to clinical and radiological predictors for counselling families and for early identification of preterm infants at risk of NDI. The findings of our study may act as adjunct to clinical information to aid clinical decision making and guide the care of preterm infants. The goal is to enhance prediction of an infant’s probability of developing NDI given his/her set of predictors (precision medicine), so that referral to early interventions and rehabilitation, known to improve outcomes, can be initiated timely.

Future research should continue to explore the role of ML in prediction of neonatal outcomes using clinical variables and existing datasets. Tools should be developed to implement the use of ML models at the bedside by developing clinical calculators of the probability of developing NDI and transforming prediction models into risk-based scores and algorithms. The clinical calculators derived from other prediction models have been widely used in perinatal practice to aid for counselling families or when critical decisions are discussed and can be made available for use by clinicians at hand.

## Figures and Tables

**Table 1:** Population Characteristics of Very Preterm Infants Stratified by Neurodevelopmental Status.

	No NDI (N = 468)	NDI (N = 195)	Overall (N = 663)
Maternal and Prenatal Variables			
Maternal Age, mean (SD)	30 (5.5)	29 (5.8)	30 (5.6)
Single parent	51/457 (11)	33/191 (17)	84/648 (13)
Hollingshead SES			
Class I	47/414 (10)	14/164 (7)	61/578 (9)
Class II	161/414 (34)	46/164 (24)	207/578 (31)
Class III	122/414 (26)	40/164 (20)	162/578 (24)
Class IV	62/414 (13)	37/164 (19)	99/578 (15)
Class V	22/414 (5)	27/164 (14)	49/578 (7)
Maternal smoking	121/451 (26)	62/ 183 (32)	183/634 (28)
Maternal substance use	34/468 (7)	24/195 (12)	58/663 (9)
Maternal diabetes	440/468 (94)	176/195 (90)	616/663 (93)
Maternal hypertension	90/468 (19)	31/195 (16)	121/663 (18)
Perinatal Variables			
Chorioamnionitis	58/468 (12)	33/195 (17)	91/663 (14)
Optimal antenatal steroids (<7 days)	174/468 (37)	48/195 (25)	222/663 (33)
Intrapartum magnesium sulphate	196/468 (42)	69/195 (35)	265/663 (40)
Cesarean delivery	270/468 (58)	122/195 (63)	392/663 (59)
Gestational age in weeks, mean (SD)	28 (1.7)	27 (2)	28 (2)
z-scores of birth weight, mean (SD)	0.013 (0.82)	−0.081 (0.88)	−0.015(0.84)
Infant male sex	241/468 (51)	123/195 (63)	364/665 (55)
5-min Apgar score, median (IQR)	8 [6, 9]	7 [6, 8]	7 [6, 9]
Small for gestational age (<10th centile by Kramer)	38/468 (8)	17/195 (9)	55/663 (8)
Singleton	304/468 (65)	134/195 (69)	438/663 (66)
Outborn status	32/468 (7)	16/195 (8)	48/663 (7)
Neonatal Variables			
BPD (oxygen at 36 weeks)	95/425 (20)	73/188 (37)	168/613 (25)
Severe IVH (grade 3,4)	19/468 (4)	41/195 (21)	60/663 (9)
WMD (periventricular leukomalacia or porencephaly)	6/468 (1)	33/195 (17)	39/663 (6)
Neonatal sepsis/systemic infection	116/468 (25)	77/195 (39)	193/663 (29)
Severe ROP ³ stage 3	30/468 (6)	38/195 (19)	68/663 (10)
Medically treated ductus arteriosus	148/468 (32)	82/195 (42)	230/665 (35)
Cardiopulmonary resuscitation	18/468 (4)	33/195 (17)	51/663 (8)
Inotropes	36/468 (8)	64/195 (33)	100/663 (15)
Systemic steroids (Dexamethazone)	53/468 (11)	55/195 (28)	108/663 (16)
Major surgery	28/468 (6)	36/195 (18)	64/668 (10)
Inhaled nitric oxide	17/468 (4)	31/195 (16)	48/668 (7)
Mechanical ventilation (days)			
None (or no information)	134/468 (29)	39/195 (20)	173/668 (26)
1–7	208/468 (35)	57/195 (29)	265/663 (40)
7–28	65/468 (14)	31/195 (16)	96/663 (14)
>28	61/468 (13)	68/195 (35)	129/663 (19)
Total length of stay (days), Median (IQR)	68 [51, 90]	89 [59, 126]	70 [53, 101]

Data are presented as n/N (percentage), mean (SD) or median (IQR). Abbreviations: BPD = bronchopulmonary dysplasia, IQR = interquartile range, IVH = intraventricular hemorrhage, NDI =neurodevelopmental Impairment, ROP= retinopathy of prematurity, SD = standard deviation, SES = socioeconomic status, WMD = white matter disease.

**Table 2:** Diagnostic Properties of Prediction Models of Neurodevelopmental Impairment in Very Preterm Infants.

	Accuracy	Sensitivity	Specificity	PPV	NPV	F	AUC (95% CI)
Logistic regression	0.82	0.54	0.93	0.78	0.83	0.64	0.73 (0.62, 0.83)
Elastic net regression	0.79	0.64	0.86	0.66	0.85	0.65	0.74 (0.64, 0.85)
Random forest	0.74	0.79	0.72	0.54	0.89	0.65	0.79 (0.70, 0.88)
Gradient boosting	0.69	0.79	0.64	0.48	0.88	0.60	0.74 (0.64, 0.84)

Abbreviations: AUC Area under the Receiver Operating Characteristic Curve, CI = confidence interval, PPV positive predictive value, NPV negative predictive value.

**Table 3:** Ensemble Voting for the Prediction Models of Neurodevelopmental Impairment in Very Preterm Infants (Combined).

Votes	No NDI	NDI
0	53 (91%)	5 (9%)
1	18 (78%)	5 (22%)
2	11 (73%)	4 (27%)
3	6 (55%)	5 (45%)
4	5 (20%)	20 (80%)

Abbreviations: NDI = neurodevelopmental impairment.

**Table 4:** Variable Importance Ranking of the Four Models to Predict Neurodevelopmental Impairment in Very Preterm Infants: The Ten Most Important Predictors for Each Model.

Logistic Regression		Elastic Net Regression	
Variable	Importance	Variable	Importance
Resuscitation	100	White matter disease	100
White matter disease	84	Resuscitation	53
Maternal age	77	HFOV >1 week	37
Severe birth asphyxia	64	Inotropes	31
Male infant	62	Maternal substance use	24
Maternal psychiatric disease	60	Maternal psychiatric disease	22
Intrapartum antibiotics	57	Severe (grades 3 or 4) IVH	21
Inotropes	53	Major surgery	21
Maternal substance use	47	HFOV 0–1 week	19
HFOV >1 week	44	Chorioamnionitis	14
Random Forest Ensemble		Gradient Boosting	
Variable	Importance	Variable	Importance
Maternal age	100	Maternal age	100
z-scores of birth weight	76	z-scores of birth weight	60
Invasive mechanical ventilation (hours)	47	White matter disease	22
Gestational age	31	Inotropes	17
Parity	21	Gestational age	17
Previous abortion	16	Parity	14
Inotropes	13	Intrapartum antibiotics	12
White matter disease	13	Neonatal sepsis	11
Optimal antenatal steroids	11	Resuscitation	11
Birth asphyxia	11	Optimal antenatal steroids	9

Abbreviations: HFOV = high frequency oscillatory ventilation, IVH = intraventricular hemorrhage.

## Data Availability

Due to privacy data and ethical restrictions, data are unavailable for public but can be viewed by review committee upon request and after approval of REB at IWK Health.

## Supplementary Materials

The following supporting information can be downloaded at: https://www.mdpi.com/article/10.3390/children11121512/s1, Table S1: Comparison of Four Prediction Methods: Logistic Regression, Elastic Net Regression, Random Forest and Gradient Boosting; Table S2: Candidate Predictors of Population-based Cohort of Very Preterm Infants Stratified by NDI Status.

## Abbreviations

AUC/AUROC	Area Under Receiver Operation Characteristics Curve
Bayley	Bayley scale of infant and toddler development
CA	Corrected age
CI	Confidence interval
CP	Cerebral palsy
CUS	Cranial ultrasound
EN	Elastic net regression
LR	Logistic regression
ML	Machine learning
NDI	Neurodevelopmental impairment
NPV	Negative Predictive Value
PFUP	Perinatal Follow Up Program
PPV	Positive Predictive Value
RF	Random forest ensemble
SD	Standard deviation
XGB	Gradient boost trees

## References

[1] Lui K., Lee S.K., Kusuda S., Adams M., Vento M., Reichman B., Darlow B.A., Lehtonen L., Modi N., Norman M. (2019). Trends in outcomes for neonates born very preterm and very low birth weight in 11 high-income countries. J. Pediatr..

[2] Sarda S.P., Sarri G., Siffel C. (2021). Global prevalence of long-term neurodevelopmental impairment following extremely preterm birth: A systematic literature review. J. Int. Med. Res..

[3] Adams-Chapman I., Heyne R.J., DeMauro S.B., Duncan A.F., Hintz S.R., Pappas A., Vohr B.R., McDonald S.A., Das A., Newman J.E. (2018). Neurodevelopmental impairment among extremely preterm infants in the neonatal research network. Pediatrics.

[4] Spittle A., Orton J., Anderson P.J., Boyd R., Doyle L.W. (2015). Early developmental intervention programmes provided post hospital discharge to prevent motor and cognitive impairment in preterm infants. Cochrane Database Syst. Rev..

[5] Lemmon M.E., Huffstetler H., Barks M.C., Kirby C., Katz M., Ubel P.A., Docherty S.L., Brandon D. (2019). Neurologic outcome after prematurity: Perspectives of parents and clinicians. Pediatrics.

[6] Crilly C.J., Haneuse S., Litt J.S. (2021). Predicting the outcomes of preterm neonates beyond the neonatal intensive care unit: What are we missing?. Pediatr. Res..

[7] Ranganathan P., Pramesh C.S., Aggarwal R. (2017). Common pitfalls in statistical analysis: Logistic regression. Perspect. Clin. Res..

[8] Tay J.K., Narasimhan B., Hastie T. (2023). Elastic Net Regularization Paths for All Generalized Linear Models. J. Stat. Softw..

[9] Deo R.C. (2015). Machine Learning in Medicine. Circulation.

[10] Wright M.N., Ziegler A. (2017). Ranger: A Fast implementation of random forests for high dimensional data in C++ and R. J. Stat. Softw..

[11] Chen T., He T. Xgboost: Extreme Gradient Boosting, R Package Version 1.7.8.1, 2024, 1–4. https://cran.r-project.org/web/packages/xgboost/vignettes/xgboost.pdf.

[12] Weiss L.G., Oakland T., Aylward G.P. (2010). Bayley-III Clinical Use and Interpretation.

[13] Papile L.A., Burstein J., Burstein R., Koffler H. (1978). Incidence and evolution of subependymal and intraventricular hemorrhage: A study of infants with birth weights less than 1500 gm. J. Pediatr..

[14] Mohammad K., Scott J.N., Leijser L.M., Zein H., Afifi J., Piedboeuf B., de Vries L.S., van Wezel-Meijler G., Lee S.K., Shah P.S. (2021). Consensus approach for standardizing the screening and classification of preterm brain injury diagnosed with cranial ultrasound: A canadian perspective. Front. Pediatr..

[15] Guillot M., Chau V., Lemyre B. (2020). Routine imaging of the preterm neonatal brain. Paediatr. Child. Health.

[16] Hand I.L., Shellhaas R.A., Milla S.S., Cummings J.J., Adams-Chapman I.S., Aucott S.W., Goldsmith J.P., Kaufman D.A., Martin C.R., Committee on Fetus and Newborn, Section On Neurology, Section On Radiology (2020). Routine neuroimaging of the preterm brain. Pediatrics.

[17] Youden W.J. (1950). Index for rating diagnostic tests. Cancer.

[18] Kuhn M. (2008). Building predictive models in R using the caret package. J. Stat. Softw..

[19] Christodoulou E., Ma J., Collins G.S., Steyerberg E.W., Verbakel J.Y., Van Calster B. (2019). A systematic review shows no performance benefit of machine learning over logistic regression for clinical prediction models. J. Clin. Epidemiol..

[20] van Boven M.R., Henke C.E., Leemhuis A.G., Hoogendoorn M., van Kaam A.H., Königs M., Oosterlaan J. (2022). Machine learning prediction models for neurodevelopmental outcome after preterm birth: A scoping review and new machine learning evaluation framework. Pediatrics.

[21] Baker S., Kandasamy Y. (2023). Machine learning for understanding and predicting neurodevelopmental outcomes in premature infants: A systematic review. Pediatr. Res..

[22] Juul S.E., Wood T.R., German K., Law J.B., Kolnik S.E., Puia-Dumitrescu M., Mietzsch U., Gogcu S., Comstock B.A., Li S. (2023). Predicting 2-year neurodevelopmental outcomes in extremely preterm infants using graphical network and machine learning approaches. EClinicalMedicine.

[23] Routier L., Querne L., Ghostine-Ramadan G., Boulesteix J., Graïc S., Mony S., Wallois F., Bourel-Ponchel E. (2023). Predicting the neurodevelopmental outcome in extremely preterm newborns using a multimodal prognostic model including brain function information. JAMA Netw. Open.

[24] Chung H.W., Chen J.C., Chen H.L., Ko F.Y., Ho S.Y., Taiwan Premature Infant Follow-Up Network (2024). Developing a practical neurodevelopmental prediction model for targeting high-risk very preterm infants during visit after NICU: A retrospective national longitudinal cohort study. BMC Med..

[25] Collins G.S., Moons K.G.M., Dhiman P., Riley R.D., Beam A.L., Van Calster B., Ghassemi M., Liu X., Reitsma J.B., van Smeden M. (2024). TRIPOD+AI statement: Updated guidance for reporting clinical prediction models that use regression or machine learning methods. BMJ.

