# Supplementary material for: Prediction of Neurodevelopmental Outcomes in Very Preterm Infants: Comparing Machine Learning Methods to Logistic Regression
Source: Children (Basel). 2024 Dec 12;11(12):1512. doi: 10.3390/children11121512 (PMC11674291; doi:10.3390/children11121512)
Supplement: Supplementary file 1 [file children-11-01512-s001.zip › children-3338189-supplementary.pdf]

**Supplementary Table S1: Comparison of Four Prediction Methods: Logistic Regression, Elastic Net Regression, Random Forest and Gradient Boosting**

| | **Logistic Regression** | **Elastic Net** | **Random Forest** | **Gradient Boosting** |
|---|---|---|---|---|
| Type | Linear Model | General Linear Model with Regularization | Ensemble Learning (Bagging) | Ensemble Learning (Boosting) |
| Application | Binary Classification | Regression and Classification | Regression and Classification | Regression and Classification |
| Strengths | Simple, interpretable, fast | Handles multicollinearity, feature selection | Handles nonlinear relationships, robust to noise | Handles complex and nonlinear relationships, scalable, efficient with large datasets |
| Weaknesses | Limited to linear relationships | Performance depends on hyperparameters selected | May overfit if trees are deep | Complex to tune, higher memory usage |
| Interpretability | High (coefficient) | High (regularized coefficient) | Low (feature importance) | Low (feature importance) |
| Overfitting Risk | Low (if regularized) | Medium | Medium (with tuning) | Low |
| Hyperparameters | none | Alpha, Lamda ratio | Number of trees, depth, features sampled | 10+, (e.g. Learning rate, max depth, tree estimators,.. ) |
| Regularization | none applied | Combination of L1 and L2 (weighted) | None | Built-in (regularization for boosting) |
| Feature Selection | No | Yes (by regularization) | No | No |
| Missing Data Handling | Not supported | Not supported | Can handle by surrogate splits | Can handle internally |
| Computation Time | Very fast | Fast | Medium | Slow (for large datasets without tuning) |

**Supplementary Table S2: Candidate Predictors of Population-based Cohort of Very Preterm Infants Stratified by NDI Status**

| | No NDI (N=468) | NDI (N=195) | Overall (N=663) | Missing |
|---|---|---|---|---|
| **Maternal & Prenatal Variables (n=13)** | | | | |
| Age of mother in years, mean (SD) | 30.0 (5.54) | 28.7 (5.81) | 29.6 (5.65) | |
| Mother's BMI, mean (SD) | 26.4 (6.58) | 26.7 (7.39) | 26.5 (6.82) | 43/663 (7%) |
| Parity of mother, mean (SD) | 0.635 (0.5) | 0.779 (1.15) | 0.68 (1.01) | |
| Previous abortions, mean (SD) | 0.549 (0.95) | 0.528 (0.90) | 0.54 (0.93) | |
| Socioeconomic status Class I | 47/414 (11.4%) | 14/164 (8.5%) | 61/578 (10.6%) | 85/663 (13%) |
| Class II | 161/414 (38.9%) | 46/164 (28.1%) | 207/578 (35.8%) | |
| Class III | 122/414 (29.5%) | 40/164 (24.4%) | 162/578 (28.0%) | |
| Class IV | 62/414 (15.0%) | 37/164 (22.6%) | 99/578 (17.1%) | |
| Class V | 22/414 (5.3%) | 27/164 (16.5%) | 49/578 (8.5%) | |
| Maternal smoking | 121/451 (26.8%) | 62/183 (33.9%) | 183/634 (28.9%) | 29/663 (4%) |
| Maternal substance use | 34/468 (7.3%) | 24/195 (12.3%) | 58/663 (8.7%) | |
| Maternal antidepressants | 43/468 (9.2%) | 16/195 (8.2%) | 59/663 (8.9%) | |
| Maternal psychiatric disorder | 73/468 (15.6%) | 44/195 (22.6%) | 117/663 (17.6%) | |
| Maternal diabetes | 440/468 (94.0%) | 176/195 (90.3%) | 616/663 (92.9%) | |
| Maternal hypertension | 90/468 (19.2%) | 31/195 (15.9%) | 121/663 (18.3%) | |
| Rural vs urban dweller | 126/468 (26.9%) | 55/195 (28.2%) | 181/663 (27.3%) | |
| Single vs dual parent family | 406/457 (88.8%) | 158/191 (82.7%) | 564/648 (87.0%) | 15/663 (2%) |
| **Perinatal Variables (n=24)** | | | | |
| Ruptured membranes > 18hrs | 106/439 (24.2%) | 31/182 (17.0%) | 137/621(22.1%) | 42/663 (6%) |
| Placental abruption | 37/468 (7.9%) | 12/195 (6.2%) | 49/663 (7.4%) | |
| Fetal distress | 32/468 (6.8%) | 17/195 (8.7%) | 49/663 (7.4%) | |
| Maternal tocolytic use | 186/468 (39.7%) | 84/195 (43.1%) | 270/663 (40.7%) | |
| Maternal group B strep status | 51/468 (10.9%) | 26/195 (13.3%) | 77/663 (11.6%) | |
| Intrapartum antibiotics | 192/468 (41.0%) | 65/195 (33.3%) | 257/663 (38.8%) | |
| Chorioamnionitis or Funisitis | 58/468 (12.4%) | 33/195 (16.9%) | 91/663 (13.7%) | |
| Intrapartum magnesium sulfate | 196/468 (41.9%) | 69/195 (35.4%) | 265/663 (40.0%) | |
| Optimal antenatal steroids (>24 hours and < 7 days) | 174/468 (37.2%) | 48/195 (24.6%) | 222/663 (33.5%) | |
| Caesarean delivery | 270/468 (57.7%) | 122/195 (62.6%) | 392/663 (59.1%) | |
| Multiples | 164/468 (35.0%) | 61/195 (31.3%) | 225/663 (33.9%) | |

| | No NDI (N=468) | NDI (N=195) | Overall (N=663) | Missing |
|---|---|---|---|---|
| Degree of birth depression | | | | |
| mild | 123/468 (26.3%) | 39/195 (20.0%) | 162/663 (24.4%) | |
| moderate | 305/468 (65.2%) | 124/195 (63.6%) | 429/663 (64.7%) | |
| severe | 40/468 (8.5%) | 32/195 (16.4%) | 72/663 (10.9%) | |
| Gestational age in weeks, mean (SD) | 28.1 (1.70) | 27.2 (2.10) | 27.8 (1.87) | |
| z-scores of weight | 0.0130 (0.825) | -0.0807 (0.880) | -0.0146 (0.842) | |
| Infant male sex | 241/468 (51.5%) | 123/195 (63.1%) | 364/663 (54.9%) | |
| 1-minute Apgar score, mean (SD) | 5.22 (2.22) | 4.56 (2.54) | 5.03 (2.33) | 6/663 (1%) |
| 5-minute Apgar score, mean (SD) | 7.28 (1.70) | 6.71 (2.10) | 7.11 (1.84) | 7/663 (1%) |
| Small for gestational age <10th centile by Kramer et al | 38/468 (8.1%) | 17/195 (8.7%) | 55/663 (8.3%) | |
| Admission temp (Celsius), mean (SD) | 36.7 (0.645) | 36.5 (0.907) | 36.7 (0.736) | 6/663 (1%) |
| Admission systolic blood pressure Mean (SD) | 50.2 (10.0) | 49.6 (10.7) | 50.1 (10.2) | 7/663 (1%) |
| Admission diastolic blood pressure Mean (SD) | 28.2 (9.51) | 28.1 (9.20) | 28.2 (9.41) | 7/663 (1%) |
| Admission mean blood pressure Mean (SD) | 35.9 (9.05) | 35.5 (8.81) | 35.8 (8.98) | 5/663 (0.8%) |
| Admission hemoglobin, mean (SD) | 163 (27.6) | 155 (27.1) | 160 (27.7) | 4/663 (0.6%) |
| Inborn vs outborn | 436/468 (93.2%) | 179/195 (91.8%) | 615/663 (92.8%) | |
| **Postnatal Variables (n=24)** | | | | |
| Surfactant therapy | 323/468 (69.0%) | 152/195 (77.9%) | 475/663 (71.6%) | |
| Bronchopulmonary dysplasia (oxygen at 36 weeks) | 95/425 (22.4%) | 73/188 (38.8%) | 168/613 (27.4%) | 50/663 (8%) |
| Sepsis/systemic infection | 116/468 (24.8%) | 77/195 (39.5%) | 193/663 (29.1%) | |
| Severe intraventricular hemorrhage (grade 3, 4 by Papile) | 19/468 (4.1%) | 41/195 (21.0%) | 60/663 (9.0%) | |
| White matter disease (porencephaly or PVL) | 6/468 (1.3%) | 33/195 (16.9%) | 39/663 (5.9%) | |
| Severe ROP (grades 3-5) | 30/468 (6.4%) | 38/195 (19.5%) | 68/663 (10.3%) | |
| Medically treated ductus arteriosus | 148/468 (31.6%) | 82/195 (42.1%) | 230/663 (34.7%) | |
| Neonatal anemia | 347/468 (74.1%) | 163/195 (83.6%) | 510/663 (76.9%) | |
| Neonatal thrombocytopenia | 77/468 (16.5%) | 62/195 (31.8%) | 139/663 (21.0%) | |
| Severe neonatal hypoglycemia (< 1.67 mml/L serum glucose) | 55/468 (11.8%) | 25/195 (12.8%) | 80/663 (12.1%) | |
| Neonatal hypoglycemia | 59/441 (13.4%) | 24/191 (12.6%) | 83/632 (13.1%) | 31/663 (5%) |
| Air leaks | 24/468 (5.1%) | 18/195 (9.2%) | 42/663 (6.3%) | |
| Systemic steroids (Dexamethasone) | 53/468 (11.3%) | 55/195 (28.2%) | 108/663 (16.3%) | |
| Lowest blood pressure during the first 24 hours, Mean (SD) | 28.4 (4.84) | 27.3 (5.07) | 28.1 (4.93) | 3/663 (0.5%) |
| Ductus surgery | 29/468 (6.2%) | 34/195 (17.4%) | 63/663 (9.5%) | |

| | Normal (N=468) | NDI (N=195) | Overall (N=663) | Missing |
|---|---|---|---|---|
| Inotropes | 36/468 (7.7%) | 64/195 (32.8%) | 100/663 (15.1%) | |
| Major surgery | 28/468 (6.0%) | 36/195 (18.5%) | 64/663 (9.7%) | |
| Nitric oxide in the baby | 17/468 (3.6%) | 31/195 (15.9%) | 48/663 (7.2%) | |
| Duration of HFOV None | 440/468 (94.0%) | 144/195 (73.8%) | 584/663 (88.1%) | |
| 0-1 week | 16/468 (3.4%) | 20/195 (10.3%) | 36/663 (5.4%) | |
| >1 week | 12/468 (2.6%) | 31/195 (15.9%) | 43/663 (6.5%) | |
| Duration of mechanical ventilation (hours) None | 134/468 (28.6%) | 39/195 (20.0%) | 173/663 (26.1%) | |
| (0,24) | 93/468 (19.9%) | 24/195 (12.3%) | 117/663 (17.6%) | |
| (24,72) | 70/468 (15.0%) | 17/195 (8.7%) | 87/663 (13.1%) | |
| (72,168) | 45/468 (9.6%) | 16/195 (8.2%) | 61/663 (9.2%) | |
| (168,672) | 65/468 (13.9%) | 31/195 (15.9%) | 96/663 (14.5%) | |
| (672, Inf) | 61/468 (13.0%) | 68/195 (34.9%) | 129/663 (19.5%) | |
| Days on parenteral nutrition Mean (SD) | 26.6 (17.1) | 35.7 (24.2) | 29.3 (19.8) | 4/663 (0.6%) |
| Caffeine therapy | 431/468 (92.1%) | 182/195 (93.3%) | 613/663 (92.5%) | |
| Any cardiopulmonary resuscitation | 18/468 (3.8%) | 33/195 (16.9%) | 51/663 (7.7%) | |
| Total length of hospital stay (days) Mean (SD) | 73.3 (30.5) | 104 (74.8) | 82.4 (49.9) | 13/663 (2%) |

| **Dropped Variables (n=12)** | **No NDI (N=468)** | **NDI (N=195)** | **Overall (N=663)** | **Missing** |
|---|---|---|---|---|
| Maternal alcohol use | 1/453 (0.0%) | 3 (1.7%) | 4 (0.6%) | 28/663 (4.2%) |
| Maternal racial origin Black | 13/376 (3.5%) | 7/155 (4.5%) | 20/531 (3.8%) | 132 (19.9%) |
| White | 341/376 (90.7%) | 130/155 (83.9%) | 471/531 (88.7%) | 132 (19.9%) |
| Asian or Pacific Islander | 12/376 (3.2%) | 7/155 (4.5%) | 19/531 (3.6%) | 132 (19.9%) |
| Native North American | 5/376 (1.3%) | 6/155 (3.9%) | 11/531 (2.1%) | 132 (19.9%) |
| Other | 5/376 (1.3%) | 5/155 (3.2%) | 10/531 (1.9%) | 132 (19.9%) |
| Intrapartum fever (>38 degrees C) | 6/468 (1.3%) | 3/195 (1.5%) | 9/663 (1.4%) | |
| Oxygen at discharge home | 14/468 (3.0%) | 7/195 (3.6%) | 21/663 (3.2%) | |
| Neonatal hydrocephalus | 7/468 (1.5%) | 23/195 (11.8%) | 30/663 (4.5%) | |
| Persistent pulmonary hypertension | 2/468 (0.4%) | 3/195 (1.5%) | 5/663 (0.8%) | |
| Necrotizing enterocolitis | 12/468 (2.6%) | 12/195 (6.2%) | 24/663 (3.6%) | |
| Neonatal meningitis | 6/468 (1.3%) | 3/195 (1.5%) | 9/663 (1.4%) | |
| Pulmonary haemorrhage | 9/468 (1.9%) | 10/195 (5.1%) | 19/663 (2.9%) | |
| Ventricular access device for severe hydrocephalus | 5/468 (1.1%) | 19/195 (9.7%) | 24/663 (3.6%) | |
| Surgery or Bevacizumab for ROP No ROP | 447/454 (98.5%) | 159/163 (97.6%) | 606/617 (98.2%) | 46/663 (6.9%) |
| ROP, no treat | 7/454 (1.5%) | 4/163 (2.5%) | 11/617 (1.8%) | 46/663 (6.9%) |
| ROP treated | 0 (0%) | 0 (0%) | 0 (0%) | 46/663 (6.9%) |
